# Current status of research on the mechanisms of tumor-associated macrophages in esophageal cancer progression

**DOI:** 10.3389/fonc.2024.1450603

**Published:** 2024-11-29

**Authors:** Yuchao Tang, Tingting Shi, Shu Lin, Taiyong Fang

**Affiliations:** ^1^ Department of Gastroenterology, The Second Affiliated Hospital of Fujian Medical University, Quanzhou, Fujian, China; ^2^ Centre of Neurological and Metabolic Research, The Second Affiliated Hospital of Fujian Medical University, Quanzhou, Fujian, China; ^3^ Group of Neuroendocrinology, Garvan Institute of Medical Research, Sydney, Australia

**Keywords:** tumor-associated macrophages, esophageal carcinoma, tumor microenvironment, mechanism, clinical treatment

## Abstract

Esophageal carcinoma (EC) is one of the most common tumors in China and seriously affects patient survival and quality of life. In recent years, increasing studies have shown that the tumor microenvironment is crucial in promoting tumor progression and metastasis. Tumor-associated macrophages (TAM) are key components of the tumor immune microenvironment and promote both tumor growth and antitumor immunity. Much evidence suggests that TAMs are closely associated with esophageal tumors. However, understanding of the clinical value and mechanism of action of TAM in esophageal cancer remains limited. Therefore, we reviewed the status of research on the role and mechanism of action of TAM in EC progression and summarized its potential clinical application value to provide a theoretical basis for the clinical treatment of EC.

## 1 Introduction

Esophageal cancer (EC) is one of the most dangerous malignant diseases in the world and negatively affects human health. According to global cancer statistics, the number of new cases of EC exceeded 510,000 in 2022, while the number of deaths caused by EC reached 445,000, with incidence and mortality rates ranking eleventh and seventh among all cancers, respectively (1–3). China has a high incidence of EC (4). Despite the decline in incidence, the absolute number of new EC cases remains high, accounting for more than half of the new cases globally due to its large population base (5, 6). This indicates that the burden of EC remains high, both globally and in China.

The main histological types of EC are esophageal squamous cell carcinoma (ESCC) and esophageal adenocarcinoma (EAC). The two histological subtypes differ significantly in terms of the tumor site, etiology, and prognosis. ESCC occurs predominantly in the stratified squamous epithelium of the upper two-thirds of the esophagus, whereas EAC usually occurs in the lower one-third of the esophagus and the esophagogastric junction and originates predominantly from Barrett’s mucosa (7). Chronic smoking and heavy alcohol consumption are the most important risk factors for ESCC. Overheated diets, pro-inflammatory diets (including pickles or sauerkraut, fried foods, and red meat), and betel quid chewing are strongly associated with ESCC, and diets low in dietary fiber may increase the risk of ESCC (8–11). Meanwhile, gastroesophageal reflux is a major risk factor for EAC due to chronic inflammation of the esophagus caused by repeated irritation of the esophageal epithelium, which in turn leads to cancer. Obesity promotes EAC through mechanical and metabolic changes (12). The occurrence of ESCC and EAC was significantly related to the level of economic development in the region. In a few high-income countries and regions (e.g., the United States, Europe, and Australia), the proportion of EAC is higher, with a clear upward trend, whereas in low-income countries and regions (e.g., sub-Saharan Africa and Asia), the proportion of ESCC is markedly higher. This may be due to an increase in the number of obese patients and those with gastroesophageal reflux disease (GERD) in developed countries. Studies have suggested that a reduction in chronic *Helicobacter pylori* infections may be a protective factor against EAC (13).

**Figure 1:**
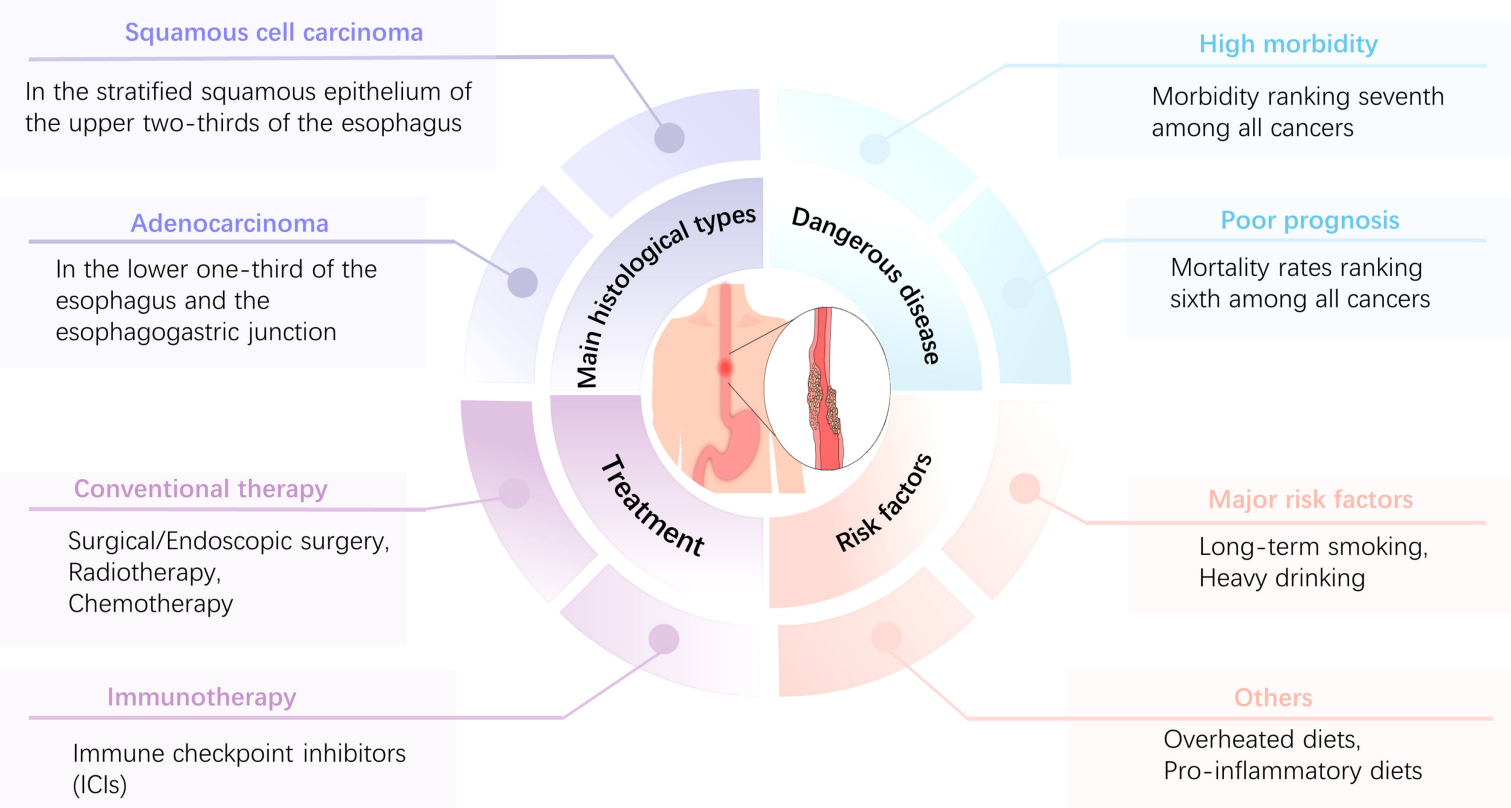
Main characteristics of EC.EC is characterized by high morbidity and a poor prognosis, with morbidity and mortality ranking seventh and sixth, respectively, among all tumors. The main histological types are ESCC and EAC, and long-term smoking and excessive alcohol consumption are the most important risk factors. Patients with esophageal cancer can be treated with surgery, radiation therapy, chemotherapy, immunotherapy, or combination therapies, depending on the stage and grade of the tumor.

To date, the treatment of esophageal cancer remains based on surgical resection of the diseased tissue and is supplemented by radiotherapy and chemotherapy (14). Moreover, it is difficult to achieve the expected results with these treatments, and they may aggravate the disease burden of patients. For example, after radical radiotherapy, esophageal anastomotic fistulas occur in a significant proportion of patients, which greatly increases their pain and seriously affects their quality of life (15, 16). Although the rapid development of endoscopic technology can effectively reduce the harm caused by EC (17), the onset of EC is characterized by an insidious, rapid progression. Most patients ignore EC in its early stages. Symptoms often become apparent (e.g., worsening of dysphagia) during the middle to late stages of the disease, when the optimal treatment period has passed, leading to a highly unfavorable prognosis for EC (18). **Figure 1** illustrates the characteristics of EC. Immunotherapy is a promising treatment option for EC. Immune checkpoint inhibitors (ICIs) prevent tumors from evading surveillance by the immune system and stimulate T-cell-mediated immune responses to kill malignant cells. ICIs, such as monoclonal antibodies to programmed cell death 1 (PD-1) and programmed cell death ligand 1 (PD-L1) (19), have been widely applied. Combined EC and neoadjuvant chemotherapy can effectively improve the survival rate and quality of life of patients (20, 21). Despite the notable value of ICIs for anticancer therapy, a significant number of patients do not respond to or develop resistance to ICIs (22, 23), which may be due to the role of tumor-associated macrophages (TAM) in the tumor microenvironment (TME).

Tumor tissues contain tumor and non-malignant cells and certain non-cellular components that constitute the TME. Non-malignant cells include fibroblasts, adipocytes, endothelial cells, and immune cells, whereas the non-cellular components include the extracellular matrix, chemokines, cytokines, and exosomes (24, 25). TAMs are a group of cells that infiltrate the tumor and its adjacent tissues and are the most numerous mesenchymal and inflammatory cells in the TME, which are closely associated with tumor progression (26–28). This paper reviewed the origin, classification, and effects of TAM on the biological behavior of EC and its possible molecular mechanisms, and discussed prospective clinical translational applications of TAM in EC.

## 2 TAMs

### 2.1 Sources of TAMs

Two main sources of macrophages exist. The first are circulating monocytes, which are derived from the differentiation of bone marrow hematopoietic stem cells. These macrophages, adapting to the ecology of local tissues and performing unique functions, form tissue-resident macrophages *in vivo* and include osteoblasts (bone tissue), Langerhans cells (skin), microglia (central nervous system), alveolar macrophages (lung tissue), and Kupffer cells (liver) (29). Another source is the yolk sac, which is formed during embryonic development. Recent studies have shown that tissue macrophages are not derived from a single source. Depending on tissue differences and developmental chronology, tissue-resident macrophages originate from three sources: early yolk sac macrophages, fetal liver monocytes, and bone marrow-derived monocytes (30, 31).

TAMs can be derived from either self-existing tissue-resident macrophages or circulating monocytes recruited from the circulation. Tissue-resident macrophages are present in the embryo and can be re-educated by the tumor to form TAMs. Alternatively, circulating monocytes can be “attracted” to the neoplastic tissue to form TAM. For example, Loyher et al. reported that TAMs in mouse lung tumors are derived from the monocyte-macrophage system and CCR2 (C-C motif chemokine receptor 2)-independent tissue-resident macrophages (32). Zhu et al. reported that the sources of TAMs in pancreatic ductal adenocarcinoma were monocytes with high expression of Ly6C (a blood-borne marker for monocytes) and embryo-derived macrophages. These two sources of TAMs have different tumors, with a reduction in the amount of TAMs in the former having a lesser effect on tumor progression, whereas a reduction in the amount of TAMs in the latter significantly slows tumor progression (33). Further in-depth studies are required to investigate differences in the molecular and biological functions of the two TAM sources.

### 2.2 Types of TAMs

TAMs have tremendous heterogeneity and plasticity. Macrophage polarization alters macrophages in response to corresponding unique microenvironmental excitations to adapt to variations in the local environment, which mainly involves manifesting different phenotypes and having specific functions (34, 35). TAM polarization can be classified into two main types: classical activation (M1) and alternating activation (M2). M1 TAMs involve mainly recognized and induced Toll-like receptor (TLR) ligands and Th1-type cytokines (e.g., IFN-γ and TNF-α) or bacterial lipopolysaccharides (LPS), and they have a characteristic CD86 phenotype, inducible nitric oxide synthase (iNOS), and MHC-II molecules, which are associated with anti-infective, inflammatory, and antitumor functions (36, 37). In contrast, M2 TAMs are mainly polarized by Th2-type cytokines (IL-4 and IL-13). Th2-type cytokines are recognized by their surface markers (CD206, CD63, and IL-10) and have been associated with biological functions such as antiparasitism, the inhibition of inflammatory responses, the promotion of organismal repair, and the promotion of tumor development. Among them, M2 can be further classified into four subtypes: M2a (wound-healing macrophages), M2b (regulatory macrophages), M2c (acquired inactivated macrophages), and M2d (narrowly defined TAM) (38). The relevant activators, molecular markers, and their functions are summarized in **Table 1**. The concept of M2 subtypes remains poorly understood. Many studies do not distinguish between M2 macrophages. In the literature, “M2” usually refers to a broad class of pro-tumorigenic TAM and equates TAM with M2 macrophages.

**Table 1:** Activators, expressed markers, and their functions in various types of macrophages.

Phenotypes of macrophages		Activator	Marker	Function	Reference
M1		LPS, IFN-γ, TNF-α	CD80, CD86, iNOS	Anti-infective, anti-tumor	(38, 99, 100)
M2	M2a	IL-4, IL-13	CD206, IL-1R, IL-10	Anti-inflammatory, promoting tissue repair	(37, 38, 101, 102)
	M2b	TLR ligand, IL-1β	IL-10, CCL1, CD86	Anti-inflammatory, tumor-promoting	(37, 38, 103)
	M2c	Glucocorticoid, IL-10, TGF-β	CD163, TLR1, TLR8	Immunosuppression, tissue remodeling	(37, 102–105)
	M2d	IL-6	IL-10, TGF-β, VEGF	Pro-tumor metastasis, angiogenesis	(37, 38, 106–108)

However, this simple TAM dichotomy has increasingly been shown to be poorly adapted to current scientific research and clinical development and has hindered the understanding of the molecular and functional diversity of TAMs. Aziz et al. reported that M1- and M2-related genes are frequently expressed in the same cells and are positively correlated. These results challenge the traditional model of macrophage polarization, which suggests that gene expression between M1 and M2 differ (39). Chiara et al. reported that extracellular vesicles/exosomes of M2-type TAM showed immune-promoting properties (40). Moreover, high levels of CD204 (+) M2 TAM infiltration in the mesenchyme or tumor were correlated with a good prognosis for patients (41). This phenomenon could be due to the complex functional properties of macrophages and the lack of nuance in dichotomies.

With the development of research techniques, several methods have been explored to analyze TAM diversity, such as single-cell transcriptomics, epigenomics, and metabolic and spatial genomics. In general, macrophage diversity has been increasingly investigated, with a focus on the link between phenotype and function (i.e., the functional spectrum model) (42).

## 3 TAMs affect the biological behavior of tumor cells

**Figure 2:**
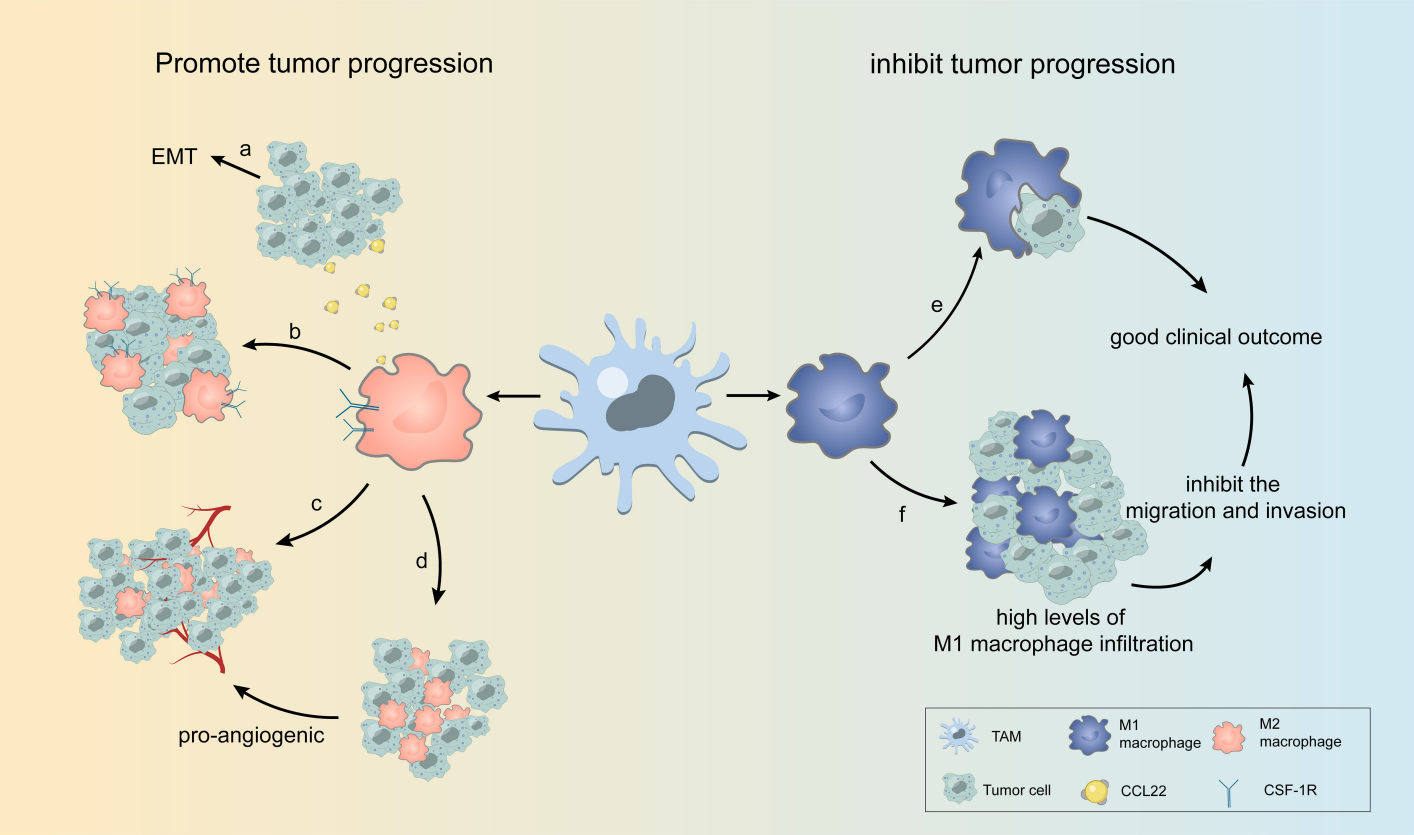
Some examples of TAMs affecting the biological behavior of tumor cells. **(A)** TAM releases CCL22, causing EMT in tumor cells. **(B)** M2 TAMs overexpressing CSF-1R are highly enriched in tumor cells. **(C, D)** In different regions of the tumor, TAM with varying amounts of blood vessels and oxygen can be divided into two groups, with one group accumulating in the perivascular region to support tumor growth and the other in the avascular or hypoxic region to promote angiogenesis. **(E, F)** M1 TAMs phagocytose tumor cells and inhibit tumor cell migration and invasion, leading to high levels of M1 macrophage infiltration associated with good clinical outcomes.

TAMs can affect various biological behaviors, such as the proliferation, invasion, and migration of many types of tumors, and can both promote and inhibit tumor development. **Figure 2** shows some examples of TAMs affecting the biological behavior of tumor cells.

### 3.1 TAMs promote tumor progression

TAMs can interact with tumor cells in various ways, regulating tumor cell proliferation, promoting angiogenesis, immunosuppression, immunosuppressant resistance, and other pro-tumorigenic effects through the secretion of cytokines or exosomes, which can lead to rapid tumor cell development. CD68(+) (pan-TAM marker) TAM is significantly increased in tumor tissues and significantly associated with poor patient prognosis (43). M2 TAMs produce numerous substances, such as CCL22 (C-C motif chemokine ligand 22), which promotes the migration and invasion of ESCC cells by inducing epithelial-mesenchymal transition (EMT) in EC cells (44, 45). Analyzing the Cancer Genome Atlas (TCGA) dataset of EC, Haddad et al. reported that macrophages were highly condensed. Furthermore, the colony-stimulating factor 1 receptor (CSF-1R) was overexpressed, although M1 and M2 were not distinguished. Given that CSF-1R is closely associated with M2 macrophage polarization, the importance of M2 macrophages in tumor promotion has been emphasized (46).

In addition to EC, TAM has been observed to promote tumor progression and lead to poor prognosis in various tumor tissues, including gastric cancer (47, 48), hepatocellular carcinoma (49, 50), colorectal cancer (51, 52), malignant melanoma (53), breast cancer (54), and glioma (55). The survival and prognosis of most patients with tumors are negatively correlated with the infiltration of TAMs (mainly M2) into tumor tissues (56, 57). TAMs target the natural course of the tumor and reduce the effectiveness of human interventions (e.g., chemotherapy and immunotherapy). After neoadjuvant chemotherapy, CD163(+) macrophages increase in tumor tissues, and M2 TAMs reduce the sensitivity of EC cells to cisplatin (58, 59). The pro-carcinogenic effects of TAMs in the same tumor tissue can be achieved by the division of labor (36). In different regions of the tumor, TAM with varying amounts of blood vessels and oxygen can be divided into two groups, with one group accumulating in the perivascular region to support tumor growth and the other in the avascular or hypoxic region to promote angiogenesis (60). In summary, TAMs promote tumor progression at multiple sites and types and reduce the benefits of drug therapy for patients.

### 3.2 TAMs inhibit tumor progression

Many studies have demonstrated that TAM is detrimental to the prognosis of cancer patients, but not all TAMs enhance tumor promotion. Wang et al. reported that more M1-type TAMs existed among patients with advanced esophageal cancer for whom neoadjuvant chemotherapy was effective; furthermore, M1 was closely related to anti-tumor function (61). The multinucleated giant cells in EC can phagocytose tumors, and cell polarization markers show that M1 is an antitumor M1 macrophage that correlates with a good clinical outcome (62). Through theoretical analyses, Cheng et al. reported that the presence of M1 macrophages was negatively correlated with tumor metastasis and the clinical stage of patients. Furthermore, *in vitro* experiments showed that M1 macrophages inhibited the migration and invasion of ESCC cells (63). In lung cancer, high levels of M1 macrophage infiltration are associated with increased overall survival (64). High levels of CD204(+) M2 macrophage infiltration have been reported as independent determinants of favorable clinical outcomes in patients with non-small-cell lung cancer (41). Inconsistencies exist in the prognostic role of TAMs in patients in different studies. Differences in the function of TAMs in different tissues likely exist due to the various TAM markers used in the studies, including the pan-macrophage marker CD68 and the M2-type macrophage markers CD206, CD163, and CD204. *In vitro* and *in vivo* experiments and clinical studies have demonstrated that TAMs inhibit tumor progression and that their main effects are on M1 macrophages. TAMs in tumors are mostly M2 with less M1 infiltration, and this may enhance innovations in anti-tumor therapy (65).

## 4 Potential mechanisms by which TAMs affect EC development

### 4.1 TAMs affect tumor growth through multiple signaling pathways

TAMs can affect various biological behaviors of EC through complex molecular mechanisms involving multiple metabolic pathways. A hypoxic environment is characteristic of tumor tissues. Macrophages in a hypoxic environment upregulate the expression of hypoxia-inducible factor-1α (HIF-1α) and promote the secretion of IL-8, thereby increasing the expression of PD-L1 in cancer cells (66). PD-1 is a membrane-surface immune inhibitory molecule widely present in activated T, B, and natural killer (NK) cells. Moreover, its main function is to inhibit T cell activation and proliferation by binding to its ligands, PD-L1 or PD-L2, effectively hindering the anti-tumor immune response.

**Figure 3:**
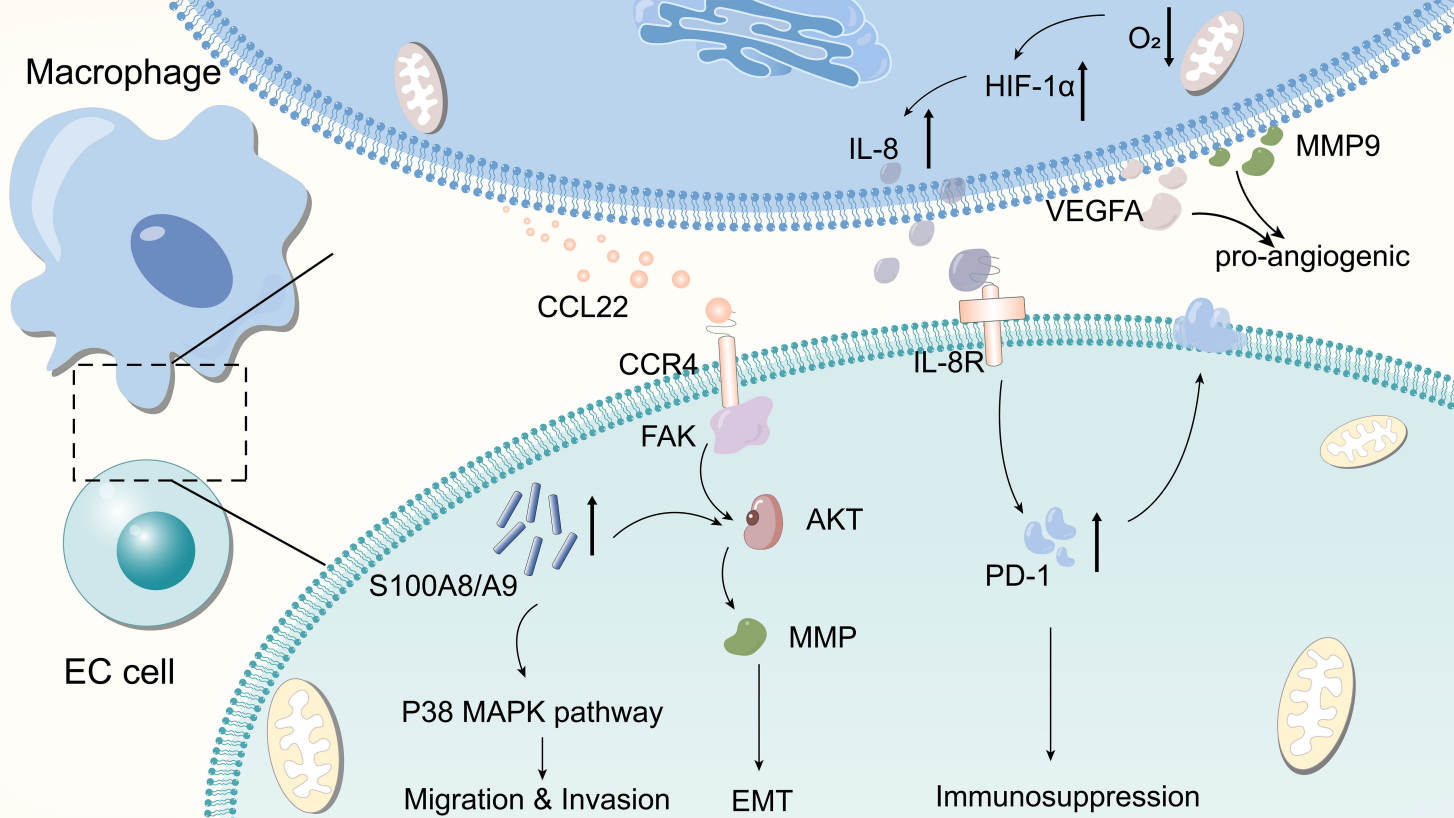
Few molecular mechanisms by which TAM affects EC.TAM releases CCL22 to bind to CCR4, activating the FAK/AKT signaling pathway and causing tumor cells to undergo EMT. Macrophages in hypoxic environments upregulate the expression of HIF-1α and promote the secretion of IL-8, the latter of which increases the expression of PD-L1 in cancer cells, which in turn promotes immunosuppression. TAM promotes the expression of S100A8/A9 in ESCC cells, which in turn enhances tumor cell migration and invasion by inducing AKT and p38 MAPK signaling pathways. TAMs can secrete pro-vascular factors such as VEGFA and MMP9 to promote tumor angiogenesis.

Metastasis is one of the hallmark activities of malignant tumors, one of the most important factors contributing to the poor prognosis of cancer, and a prerequisite for cancer cell invasion and tumor metastasis (67). TAMs promote the progression of malignant tumors by releasing multiple cytokines and chemokines. For example, CCL22 released by TAMs binds to its receptor CCR4 and triggers the activation of the FAK/AKT signaling pathway, which activates EMT in ESCC cells. This process is accompanied by the secretion of matrix metallopeptidases that contribute to the degradation of the basement membrane, thereby facilitating tumor cell invasion (44). Zhou et al. reported that MCP2 was able to cause EMT in ESCC by activating the NF-κB signaling pathway (68). TAM promotes the expression of S100A8/A9 in ESCC cells, which in turn facilitates the migration and invasion of cancer cells by inducing the AKT and p38MAPK signaling pathways (69). **Figure 3** illustrates the specific underlying mechanisms of the EC-TAM interrelationships. These complex networks of cellular interactions reveal the role of TAM in regulating the TME and provide new perspectives for understanding the molecular mechanisms underlying tumor invasion and metastasis.

The promotion of angiogenesis—involving the formation of new blood vessels that enable tumor cells to obtain more nutrients, which is conducive to tumor proliferation—is a key characteristic of tumors. Several studies have shown that TAMs are crucial in promoting tumor angiogenesis. Shou et al. observed that the expression of vascular endothelial growth factor A (VEGFA) and MMP9 in the supernatant of M2-polarised macrophages was significantly higher than that in the control group, suggesting that TAMs secrete the pro-angiogenic factors VEGFA and MMP9 and promote tumor angiogenesis (70).

### 4.2 TAMs may undermine the efficacy of checkpoint immunotherapy

PD-1/PD-L1 receptor blockers are among the most effective ICI drugs with few side effects, making them ideal targeted anticancer drugs. PD-1/PD-L1 monoclonal antibodies prevent effector T cells from losing their function by inhibiting the binding of the immunosuppressive molecule PD-1 to PD-L1. Most patients do not respond to or develop resistance to ICIs, and the exact mechanism is multifaceted, with TAMs playing an important role. Researchers have reported that TAM secretes LINC02096, the long non-coding RNA, into ESCC cells. LINC02096 binds to MLL1 and prevents ASB2-mediated ubiquitination, which enhances MLL1 stability and upregulates PD-L1/IDO-1 expression, leading to the reduced efficacy of ICIs (22, 71).

### 4.3 Intratumoral microbiomes may affect the formation of TAMs and, thus, esophageal carcinogenesis

Bacteria are present in many types of tumors, and intratumoral bacteria are predominantly found in cancer and immune cells (72). Similar to the promotional effect of gut microbiota on colon tumors, the presence of intratumoral microbes is associated with tumor progression. In analyzing the intratumoral microbial abundance of 98 patients with EC, Zhang et al. reported that patients with low intratumoral microbial diversity had significantly longer overall survival than those with highly diverse microbiota and that intratumoral microbial abundance was positively correlated with PD-L1(+) TAM (73). This study discusses information and insights regarding the design of effective anticancer treatment strategies.

### 4.4 Crosstalk between TAMs and non-malignant cells affects tumor progression

The TME is a complex ecosystem comprising tumor cells, nonmalignant cells, and the metabolites they produce. TAMs can influence tumor development through interactions with nonmalignant cells such as fibroblasts, dendritic cells, lymphocytes, and adipocytes. Hyaluronic acid secreted by cancer-associated fibroblasts (CAFs) leads to the pro-tumorigenic activity of TAMs. In addition, lactic acid secreted by CAFs contributes to the M2-type polarization of TAMs (74). TAMs are recruited to the tumor surroundings by transforming growth factor-β (TGF-β) secreted by tumor cells. At this stage, TAMs exhibit enhanced phagocytosis and inhibit the antigen-presentation function of dendritic cells by removing dead tumor cells, thereby preventing dendritic cells from contacting tumor cell antigens (75). TAMs express human leukocyte antigen (HLA) molecules, such as HLA-C, HLA-E, and HLA-G, which inhibit the activation of NK cells and T cells (76). CD8 T cells exhibit poor migration ability due to the long-term effects of TAMs, resulting in reduced infiltration into tumor nests. When TAMs are depleted, the infiltration of CD8 T cells into tumor nests can be restored, and with improvement of the efficacy of anti-tumor immunotherapy drugs (77). Adiponectin (APN) produced by adipocytes induces M2 macrophage polarization via AMP-activated kinase, whereas in the absence of APN, TAMs exhibit an M1-like phenotype (78). TAMs also modulate the components of the ECM, with effects that are mainly tumor-promoting (79). Overall, the interactions between TAMs and other components in the TME play a significant role in tumor development.

### 4.5 Effect of esophageal carcinoma cells on TAMs

Tumor cells alter their surroundings by secreting certain substances or producing metabolites to improve their survival, and TAMs have become one of their targets. Cancer cells can hijack the function of TAM by secreting small molecules of interest. In tumor tissues, malignant cells are characterized by rapid growth and a different energy metabolism system from normal cells. To maintain the rapid proliferation of cancer cells, they consume copious energy, which is mainly obtained through the anaerobic glycolysis pathway, leading to increased lactic acid levels in the TME, which in turn creates an acidic environment (80). *In vitro* experiments have demonstrated that lactate can promote TAM M2-type polarization, which in turn stimulates the growth of EC tumor cells. The introduction of lactate inhibitors reverses the stimulatory effects of lactate on macrophage polarization. More in-depth studies have suggested that lactate may promote tumor growth by affecting the AKT/ERK signaling pathway to regulate the polarization state of TAM (81). ESCC cells upregulate S100A7 protein expression to promote macrophage M2 polarization and synergize with macrophages to promote tumor growth (82). EC communicates with various cells in the TME by secreting exosomes. Exosomes from ESCC cells can induce M2 macrophage polarization by inhibiting PTEN and activating the PI3K/AKT signaling pathway, which, in turn, promotes neovascularization (70). HMGB1 is associated with hypoxia-induced macrophage polarization and is overexpressed in various tumors (83). Li et al. reported that exosomal HMGB1 isolated from an ESCC cell line promoted monocyte polarization. Furthermore, polarized macrophages exhibited M2-like features, and macrophage differentiation was inhibited when exosome release inhibitors were added (84). Song et al. demonstrated that ESCC could deliver miR-21-5p to macrophages via secreted exosomes and that miR-21-5p induced macrophage M2 polarization and promoted EC cell EMT. These findings reveal a potential regulatory mechanism, suggesting that the exosome-mediated miR-21-5p-associated signaling pathway may be crucial in regulating macrophage activity, cell polarization, and EC development in cancer cell EMT (85). These findings support further understanding of the molecular mechanisms underlying ESCC.

## 5 Clinical application of TAMs in esophageal carcinoma

### 5.1 Value of TAMs in the diagnosis and prognosis prediction of EC

TAMs can express various specific molecular markers on their surface. These markers can be used for screening and early detection of tumors to improve therapeutic efficacy, guide patient follow-up, and validate the efficacy of therapeutic agents using the characteristics of these molecular expressions. The relationship between TAM and EC is complex, and many studies have explored the potential molecular targets of EC to determine the relationship between these molecular substances and clinical outcomes in patients. **Table 2** shows the relationship between TAM and the prognosis of patients with EC, as described in the relevant literature. For example, M1-type TAM and CD56dim NK cells can be used to predict the efficacy of Camrelzumab; TREM2(+) TAM infiltration in ESCC can predict patient prognosis; and the expression of heat shock factor 1 (HSF1) in TAM can be used as a prognostic marker for ESCC (61, 86, 87). However, the values of the molecular markers vary among different studies. In a study by Li et al., CD68(+) TAM infiltration was associated with poor patient prognosis, whereas Wang et al. noted prolonged overall survival with CD68(+) TAM infiltration. These variations may again be due to the use of pan-cancer markers or differences in patient populations (88, 89).

**Table 2:** Studies of the correlation between TAM and the prognosis of patients with EC.

Study Result	Expression in TAMs	Sample Size (Case)	Ref
High levels of HSF1 expression are associated with M2 macrophage infiltration, can promote tumor proliferation, and are linked to poor prognosis.	HSF1	134	(86)
Characteristics of TREM2(+) TAMs: 1. Functionally, they are more similar to M2-type TAMs; 2. They are involved in the activation of the complement system, leading to the disruption of the cancer immunity cycle; 3. They upregulate PD-L1 on the surface of TAMs, thereby reducing the anti-tumor effects of T cells; 4. They highly express soluble factors, making them an ideal target for liquid biopsy.	TREM2(+)	67	(87)
Higher M1/M2 ratios can improve overall survival by a mechanism that may enhance the tumor-killing effect of infiltrating T cells. Limitations: Small sample size.	High M1/M2 ratio	30	(61)
CD68(+) TAM showed high concordance with MMP-9 expression, and both of them were associated with poor outcomes such as lymph node metastasis and late clinical staging	CD68(+), MMP-9	200	(88)
The densities of CD86 and IL-13 in the tumor stroma were positively correlated with the patients’ postoperative overall survival time and disease-free survival time; therefore, a prognostic prediction model based on CD86 and IL-13 has been developed, and its further integration with TNM staging can provide a more accurate prediction of postoperative ESCC patients.	CD68(+)	705	(89)
SIRPα inhibits macrophage phagocytosis; moreover, high expression of SIRPα may inhibit anti-tumor immune responses, leading to a poor prognosis in ESCC.	CD163(+) M2 macrophages have high SIRPα expression.	131	(97)
PD-1-overexpressing macrophages are the major TAMs in ESCC and are significantly associated with poor prognosis, and phagocytosis by macrophages is enhanced after anti-PD-1 treatment.	PD-1 highly expressed	200	(43)
The density of CD163(+) TAM was strongly associated with adverse outcomes such as tumor invasion, lymphatic metastasis, and hematogenous metastasis, and varied in tissues from different sites, whereas CD68(+) had no effect on tumor outcomes.	CD163(+)	2292	(109)

### 5.2 Providing new therapeutic targets for EC

The treatment of EC requires consideration of the size of the tumor, number of primary foci, depth of infiltration, and presence of distant metastases. If the tumor is advanced or has metastasized, operative treatment is no longer the preferred option, and chemotherapy and immunotherapy may be more beneficial than surgery alone. As the relationship between TAM and tumors becomes clearer, more therapeutic options for targeting TAMs are being explored and are mainly classified as follows: 1) reduce macrophage recruitment into the TME to form TAM; 2) inhibit M2 macrophage differentiation, thereby reducing M2 macrophage infiltration in tumor tissues; and 3) promote the differentiation of circulating monocytes into M1 macrophages or induce the conversion of pre-existing M2 macrophages to the M1-type to increase the number of tumor-resistant-type cells and remodel the TME.

The colony-stimulating factor 1 receptor (CSF-1R) signaling pathway mainly regulates TAM production, differentiation, and activation, and the detrimental effects of TAM in tumor therapy can be counteracted by CSF-1R inhibitors. This has been demonstrated in animal experiments, where the combination of CSF-1R and PD-1 inhibitors overcame EC resistance to PD-1/PD-L1 (90). Wang et al. reported that the mechanism of action of the traditional Chinese medicine, p-hydroxycinnamaldehyde (CMSP), was related to the induction of monocytes into M1 macrophages. After CMSP treatment, the percentage of M2 macrophages in the tumor tissue decreased while the number of M1 macrophages increased; the tumor volume was significantly reduced (91). Another study reported that small molecules promoting TAM M1 polarization loaded into exosomes and modifying the surface of exosomes with IL4R-targeting peptides for targeted delivery to IL4R-expressing TAMs in tumors increased the number of M1-type TAMs. Furthermore, potent anti-tumor immunotherapeutic capabilities were noted (92).

Similar to exosomes, nanotechnology can be used to treat tumors via TAM. Han et al. loaded astragalosides into polylactic acid nanoparticles. The presence of an M2 macrophage-binding peptide on the surface of the nanoparticles enhanced tumor killing by targeting and reversing the M2 TAM to M1 (93). Furthermore, inhibition of the CD47-SIRP1α pathway, a molecule expressed on the surface of all cells and associated with a wide range of intercellular activities, is a useful anti-tumor therapeutic strategy. Normal tissue cells in the body bind to the macrophage or neutrophil surface receptor SIRP1α via CD47 to prevent these phagocytes from misinterpreting it as a “not me” signal and killing them. This mechanism is used for immune evasion by tumor cells (e.g., head and neck squamous cell carcinoma and bladder cancer) that inhibit phagocytosis by macrophages by overexpressing CD47 (94, 95). Anti-tumor therapeutic strategies targeting CD47 have been developed and applied to lung cancer patients for whom chemotherapeutic agents are ineffective and show potent tumor suppression (96). High expression of molecules associated with the CD47-Sirpα signaling pathway has been observed in ESCC tissues and was significantly correlated with deeper penetration depths into tumor tissue and lower survival (97). Monoclonal antibodies targeting CD47Sirpα could be used in the treatment of ESCC, which may enhance the therapeutic efficacy of other anti-tumor agents such as ICIs and neoadjuvant chemotherapy. These studies provide new perspectives for a more comprehensive therapeutic approach and are expected to improve the prognosis and survival of patients with EC.

### 5.3 Challenges of TAMs in clinical translation

Currently, gaps remain in our understanding of TAM, and many difficulties in its clinical application exist. First, TAM classification requires improvement. In clinical practice, different laboratories use different TAM markers. Therefore, when the same sample is evaluated, different results may be obtained. Another challenge is the need to select a highly specific marker to accurately differentiate between different TAM types. Many of the commonly used macrophage markers are also expressed in other cell types, such as iNOS, which is commonly used as a marker for M1 macrophages but is present in vascular endothelial cells and arterial wall smooth muscle cells. CD163 is expressed in M2 macrophages and a few dendritic and endothelial cells. This leads to an increased rate of false-positives. These issues can be addressed by combining various methods to analyze different TAM subpopulations that corroborate each other using different approaches (98). Furthermore, TAM has been less studied in EC compared to other tumors, hindering its clinical translation, and more molecular mechanisms are expected to be discovered. The role of macrophages may vary in different tumors and individuals, and the tumor-specific immune environment and individual differences must be carefully considered when using TAMs for treatment. Currently, most studies remain in the preclinical stage, and more clinical studies are needed to validate their efficacy and safety.

## 6 Conclusion and prospects

This literature review provided an overview of the origin and classification of TAMs, described how TAMs affect parthenogenesis and progression, and highlighted the potential molecular mechanisms of TAM-EC interactions. Macrophages within the TME are numerous, functionally complex, and affect EC development through multiple pathways. Macrophages remain crucial in promoting tumor development by stimulating the proliferation, migration, and invasion of tumor cells; promoting angiogenesis; and inducing immunosuppression to resist the antitumor effects of chemotherapeutic drugs. However, TAM can play an anti-tumor role, which can enhance the prognosis for patients. Similarly, TME-stimulated EC cells can change the polarization state of macrophages according to gene expression, exosome secretion, and other factors, enhancing their invasiveness and ability to evade killing by the immune system, enabling them to survive in unfavorable environments. In addition, this paper explored the clinical value of TAMs in the treatment of EC and elucidated that multiple therapeutic approaches using TAMs for antitumor therapy have broad developmental prospects.

However, there are some deficiencies in the research on TAMs. Firstly, current research on TAMs mainly focuses on M2-type TAMs, and the role of M2-type TAMs in tumor promotion has been relatively clear. In contrast, relatively few studies have been conducted on M1-type TAMs, and the mechanism by which M1-type macrophages exert an anti-tumor role in EC progression needs to be further explored. Secondly, the development of TAMs is a dynamic, continuous and complex process that involves the expression of multiple molecules at different stages. On the one hand, this suggests that TAMs have great potential in tumor diagnosis and prognosis prediction. On the other hand, these diverse biomolecules have also caused difficulties in the classification of TAMs. Different studies have classified TAMs in various ways, and none of these classification methods can comprehensively summarize the characteristics of TAMs, which may lead to confusion in the classification of TAMs, which is not conducive to the horizontal comparison between different studies. Thirdly, tumorigenesis is a long-term, complex process regulated by multiple factors. Current research on TAM is mostly based on molecular biology and cytological experiments, and extensive laboratory data and testing in clinical practice are lacking. Clinical trials can enable a more complete understanding of the impact of TAM treatment on patients to be obtained, including whether the treatment produces consistent results in a broader patient population and whether the treatment has potential adverse effects. This information is critical for the translation of laboratory findings into actual treatment regimens. Therefore, in the future, multi-targeting strategies in antitumor immunotherapy for TAMs may become a hot topic of discussion. Compared with conventional single-targeted therapy, multi-targeted therapeutic strategies can better address the complex TME molecular network and reduce the likelihood of inhibition by a single component being compensated by other pathways, thereby making anti-tumor immunotherapy more effective. Future studies should focus on integrating laboratory results with clinical practice to better understand and optimize the potential benefits of TAM therapy.

## Glossary

EC: esophageal carcinoma
TME: tumor microenvironment
TAM: tumor-associated macrophages
ESCC: esophageal squamous cell carcinoma
EAC: esophageal adenocarcinoma
GERD: gastroesophageal reflux disease
ICIs: immune checkpoint inhibitors
PD-1: programmed cell death 1
PD-L1: programmed cell death ligand 1
CCR2: C–C motif chemokine receptor 2
TLR: Toll-like receptor
IFN: Interferon
TGF: transforming growth factor
TNF: Tumor Necrosis Factor
VEGF: vascular endothelial growth factor
LPS: lipopolysaccharide
CD: cluster of differentiation
iNOS: inducible NO synthase
MHC: major histocompatibility complex
IL: interleukin
CCL: C-C motif chemokine ligand
EMT: epithelial-mesenchymal transition
TCGA: The Cancer Genome Atlas
CSF-1R: colony-stimulating factor 1 receptor
HIF-1α: hypoxia-inducible factor-1α
CCR: C–C motif chemokine receptor
FAK: focal adhesion kinase
MMP: Matrix metallopeptidase
MCP2: monocyte chemotactic protein 2
NF-κB: nuclear factor kappa-B
MAPK: mitogen-activated protein kinase
VEGFA: vascular endothelial growth factor A
MLL1: mixed lineage leukaemia protein‐1
ASB2: ankyrin repeat and SOCS box containing 2
IDO-1: indoleamine 2,3‐dioxygenase 1
ERK: extracellular regulated protein kinases
PTEN: Phosphatase and Tensin Homolog deleted on Chromosome 10
PI3K: Phosphoinositide 3-kinase
HMGB1: High mobility group box-1 protein
TREM2: triggering receptor expressed in myeloid cells 2
HSF1: heat shock factor 1
CMS: Cochinchinnamomordica seed
CMSP: p-hydroxycinnamaldehyde
IL4R: Interleukin 4 receptor
SIRP1α: signal regulatory protein 1α
CAFs: cancer-associated fibroblasts
TGF-β: transforming growth factor-β
HLA: human leukocyte antigen
HLA-C: human leukocyte antigen-C
HLA-E: human leukocyte antigen-E
HLA-G: human leukocyte antigen-G
APN: adiponectin
ECM: extracellular matrix
